# Case fatality rate ‎and disability of stroke in Isfahan, Iran: Isfahan stroke registry

**Published:** 2016-01-05

**Authors:** Shahram Oveisgharan, Amir Babak Ghaemmaghami, Ahmad Bahonar, Nizal Sarrafzadegan

**Affiliations:** 1 Rush Alzheimer’s Disease Center, Rush University Medical Center, Chicago, Illinois, USA AND ‎‎Iranian Center of Neurological Research, Shariati Hospital, Tehran University of Medical ‎Sciences, Tehran, Iran; 2 Iranian Center of Neurological Research, Shariati Hospital, Tehran University of Medical ‎Sciences, Tehran, Iran; 3 Isfahan Cardiovascular Research Center, Cardiovascular Research Institute, Isfahan University of Medical Sciences, Iran

**Keywords:** Stroke, Case Fatality Rate, Disability Evaluation, Epidemiology, Risk Factors

## Abstract

**Background:** Few investigators have reported case fatality and disability of Iranian patients with stroke. This study was designed to collect morbidity and case fatality data of hospitalized patients with stroke, and stroke care quality in Isfahan, Iran.

**Methods:** From 2006 to 2011, from overlapping sources (discharge diagnoses, attending physicians, and hospitalization wards), all hospitalized patients with possible strokes were enrolled in the study, their hospital records were summarized by experienced personnel and reviewed by a neurologist with stroke experience. Patients were followed by phone calls or visited to their addresses and their 28^th^ day functional status was checked by translated modified Rankin Scale (mRS). Forms and methods were derived from the World Health Organization (WHO) Monitoring Trends and Determinants in Cardiovascular Disease and STEPS projects.

**Results:** A total of 9487 patients were identified to suffer from stroke. Their ages’ mean was 68.98 ± 13.63 years, and 50.0% were females. In hospital, case fatality was 16.5% and the 28^th^ day case fatality was 25.6%. The greatest case fatality was among intracerebral hemorrhage (ICH) patients and the least among ischemic stroke (IS) ones. Case fatality was greater among female and older patients and those with the previous history of stroke. Among survivors, only 26.9% were functionally independent (mRS < 3) which was the greatest among subarachnoid hemorrhage (SAH) patients and least among ICH patients. None of the patients were admitted to specific stroke units or received thrombolytic therapy.

**Conclusion:** The hospitalized patients with stroke in Isfahan have unfavorable outcome compared with their mates in developed countries. A low quality of stroke care may be responsible, and urgent attention is needed.

## Introduction

Stroke is the second most common cause of death worldwide at the beginning of the 21^st^ century and caused approximately 10.0% of the total deaths.^1^^,^^2^ Regional differences in stroke incidence have been reported, including the existence of a stroke belt in some countries, but causes of these disparities have not yet been fully understood.^3^

One of the most sophisticated measures to reduce stroke case fatality and morbidity is stroke unit. It is an area inside of a hospital where physicians, nurses, and other assisting personnel who have high-quality training and experience in stroke management, provide care for patients. Management of acute stroke in this type of medical facility has been shown by several studies to reduce death and disability by approximately 20.0% and improve patients’ chance of recovery and independent living.^4^ Unfortunately, there are few established stroke units in Iran at the present time.

Ischemic stroke (IS) and intracerebral hemorrhage (ICH) are the two most common types of stroke. Right now, we do not have any specific treatment for ICH, and intravenous administration of tissue plasminogen activator (tPA) is the only Food and Drug Administration (FDA)-approved therapy for IS. However, few patients benefit from tPA administration because of the limited time window (first 3.0-4.5 hours after symptoms onset) in which tPA benefits outweigh its side effects (hemorrhages).^5^

Studies about stroke epidemiology in Iran are scarce. No published study has reported disability outcome of Iranian patients with stroke. Few published studies have reported case fatality among Iranian hospitalized patients with stroke, who were among the greatest reported.^6^^,^^7^ In two other cross-sectional, hospital-based studies conducted in Qom, a well-known religious city of Iran, the case fatality rate within the 1^st^ month was reported at 24.6% in 2001 and at 15.3% in 2006-2008. Although, these figures showed an apparently declining rate over time in that region but were still more than western countries’ and less than other developing countries’.^8^^-^^12^

This study was designed to collect morbidity and case fatality data of hospitalized patients with stroke, and the frequency of tPA use and hospitalization in specific stroke units in all hospitals that admit such patients in Isfahan, Iran.

## Materials and Methods

Isfahan is the third largest city in Iran. Its metropolitan population is about 2000000 inhabitants and < 15.0% of them live in rural areas. Like other parts of the country, it has a young population, with about 55.0% of the population younger than 30 years.

From 2006 to 2011, 6 hospitals were mainly admitting patients with stroke in Isfahan, and none of them had stroke unit. Three of them had specific neurology wards, and all had general Intensive Care Units (ICU). In hospitals without neurology wards, patients with stroke were mostly hospitalized in internal medicine wards.

According to the World Health Organization (WHO) definition, stroke is a clinical syndrome characterized by rapidly developing neurological symptoms and/or signs, focal and at times global [applied to patients in deep coma and those with subarachnoid hemorrhage (SAH)], with symptoms lasting more than 24 hours unless interrupted by surgery or leading to death, and with no apparent cause other than that of vascular origin.^13^ This definition includes stroke due to cerebral infarction (IS), ICH, intraventricular hemorrhage, and SAH; it excludes subdural hemorrhage, epidural hemorrhage, or ICH or infarction caused by infection or tumor. With this clinical definition, silent stroke on imaging is not considered a stroke, and imaging confirmation is not required for stroke diagnosis. Hence, the study is based on clinical diagnoses, which have been shown to be reliable.^14^

The WHO Monitoring Trends and Determinants in Cardiovascular Disease^7^^,^^14^ project is related to events, not persons. Events are classified as first or recurrent and as fatal or nonfatal. A period of 28-day was used to define the case fatality rate and to distinguish between events. Diagnostic criteria were applied to symptoms, clinical findings and investigations undertaken within 28 days of onset. Transient ischemic attacks and events associated with trauma, blood disease or malignancy were not included.^14^ Events were categorized as “definite stroke,” “not stroke” or “unclassifiable.” Only definite stroke events which fulfilled the criteria when the available information permitted a clinical diagnosis were included in the study.

Procedures are different according to how events are identified and registered: based on their admission to hospital “hot pursuit” or by utilization of post-discharge records to obtain patient’s information retrospectively “cold pursuit”.^15^^,^^16^ A cold pursuit method has been used in this study: records of patients, who were hospitalized in either neurology or other departments under the complete or partial supervision of neurologists in Isfahan hospitals, were evaluated for possible signs and symptoms of stroke events. Search for potential stroke records was done by overlapping methods: looking through discharge diagnoses (stroke, cerebrovascular accident, ICH, SAH, vertebrobasilar insufficiency (VBI), cerebral venous thrombosis, and transient ischemic attack); looking through records by name of attending, and looking through records by wards of hospitalizations. Apart from the main mentioned 6 hospitals, patients who possibly had stroke during hospitalization and were discovered by surveillance department personnel [myocardial infarction (MI) surveillance unit] were included in the registry. All potential stroke records were evaluated by one experienced health personnel member who has continuously been under education in this regard. She summarized proper records in special checklists which were evaluated by a stroke fellow (one of the authors) to see if stroke was the diagnosis, and to determine stroke type (IS, ICH, SAH, unknown). Because Isfahan city is the center of Isfahan province, its hospitals have a referral from other cities of the province. 

All hospitalized patients who were discharged alive were followed by their address or by telephone. The patients or their close family members were asked about the patients’ health status. If a patient had died during the first 28 days after the event, a death scenario was questioned, and she/he was pretend to have died because of stroke only if other etiologies, such as a motor vehicle accident could be ruled out. In the follow-up interview, patients’ functional status was questioned by use of translated modified Rankin Scale (mRS).^17^

The term “stroke hospital admission rate” refers to both first and recurrent events. T-test was used for comparison of means, and chi-square test was used for comparison of proportions. Multiple regression was used to control confounding and extraneous variables’ effects. All the analysis was done with SPSS software (version 20, SPSS Inc., Chicago, IL, USA).

## Results

A total number of 10191 patients with primary diagnoses of stroke were recorded from 2006 to 2011. However, only 9487 (93.1%) patients met our stroke diagnostic criteria, and the rest were either labeled with other diagnosis (4.6%) or stayed unknown (2.3%). 9446 (99.6%) of patients were hospitalized and managed in the main 6 hospitals. Stroke subtypes were classified by the results of computed tomography (CT scan) done on patients presenting with stroke symptoms. Stroke verified cases constitute the sample of further analysis.

Patients’ demographic data are summarized in table 1. More than 50.0% of patients were males; mean age was 68.98 ± 13.63 around 69 with the youngest 13 and the oldest 114 years old. While SAH patients were the youngest group with their mean age about 14 years less than total average, patients with IS were the oldest ones. More than 85.0% of them were coming from urban areas, especially Isfahan city. Stroke subtypes are also shown; 79.6% of stroke events were of the ischemic type.

In hospital, case fatality rate for all types of stroke was 16.5%. Table 2 shows potential confounding factors’ effect on in hospital case fatality. Stroke subtype significantly affected in hospital case fatality rate (P < 0.001), with the greatest rate (33.9%) among patients with ICH. Patients who got dead were about 3 years older (P < 0.001), and death happened a little, although statistically significant, more in females (P = 0.042). Likewise, the rate was more among subjects with positive stroke history (P = 0.002). When all the above variables were put in a logistic regression (Table 3), sex was not further a significant risk factor of in hospital case fatality rate. The strongest risk factor was stroke subtype. 

The follow-up was successful in 83.0% of cases in different years. Compared with patients with successful follow-up, missed patients were a younger (66.82 ± 14.29 vs. 69.44 ± 13.45, P < 0.001), were less positive in history of stroke (23.2 vs. 26.7%, P = 0.004), and had the same sex frequencies (50.6 vs. 48.0% were female, P = 0.006). Furthermore, missing was less seen among ICH patients (15.0%) than among ischemic (17.7%) or SAH (18.6%) ones (P = 0.003). 

**Table 1:** Stroke subtypes and patients demographic data in Isfahan hospitalized patients with stroke, from 2006 to 2011

**Variable**	**Stroke**			**Subtypes**
	**Ischemic**	**ICH**	**SAH**	
Total [n (%)]	7548 (79.6)	1628 (17.2)	183 (1.9)	9487 (100)
Sex [n (%)]				
Male	3893 (51.7)	838 (51.6)	76 (41.8)	4881 (51.4)
Female	3639 (48.2)	787 (48.4)	106 (58.2)	4586 (48.3)
Age (years) (mean ± SD)	69.77 ± 13.12	66.75 ± 14.52	54.96 ± 16.65	68.98 ± 13.63
Min	14	13	18	13
Max	114	110	113	114
Settlement [n (%)]				
Urban				8089 (86)
Isfahan city	4820 (64.3)	893 (55.4)	99 (55.3)	5916 (62.9)
Other cities	1658 (22.1)	449 (27.8)	51 (28.5)	2173 (23.1)
Rural				1322 (14)
Isfahan city area	341 (4.6)	77 (4.8)	6 (3.4)	426 (4.5)
Other cities areas	673 (9.0)	194 (12.0)	23 (12.8)	896 (9.5)

Figures in parentheses indicate percentages.

ICH: Intracerebral hemorrhage; SAH: Subarachnoid hemorrhage; SD: Standard deviation

**Table 2:** Potential confounding factors’ effect on case fatality rate among Isfahan stroke hospitalized patients, from 2006 to 2011

**Variable**	**In hospital**	**P**	**28** ^th^ **-day**	**P**
All stroke subtypes [n (%)]	1534 (16.3)	< 0.001	1984 (25.6)	< 0.001
Ischemic	940 (12.5)		1318 (21.2)	
ICH	551 (33.9)		620 (44.8)	
SAH	43 (23.5)		46 (30.9)	
History of previous stroke [n (%)]		0.002		0.001
Negative	1052 (15.5)		1363 (24.3)	
Positive	438 (18.2)		578 (28.2)	
Sex [n (%)]		0.042		0.006
Male	758 (15.6)		986 (24.2)	
Female	784 (17.2)		1013 (27.0)	
Age (year) (mean ± SD)		< 0.001		< 0.001
Alive	68.39 ± 13.68 (n = 7881)		68.08 ± 13.43 (n = 5831)	
Dead	71.96 ± 13.06 (n = 1542)		73.37 ± 12.71 (n = 1999)	

ICH: Intracerebral hemorrhage; SAH: Subarachnoid hemorrhage; SD: Standard deviation

**Table 3:** Multiple logistic regressions of in hospital and 28 days case fatality odds on potential risk factors

**Variable**	**In hospital case fatality**			**28 days case fatality**		
	**OR**	**P**	**95% CI of OR**	**OR**	**P**	**95% CI of OR**
Age (centered on 70)	1.03	< 0.001	1.02-1.03	1.04	< 0.001	1.04-1.05
Female sex	1.11	0.09	0.98-1.24	1.14	0.018	1.02-1.27
Type of stroke		< 0.001			< 0.001	
SAH versus ischemic	3.40	< 0.001	2.37-4.89	3.13	< 0.001	2.14-4.56
ICH versus ischemic	4.04	< 0.001	3.55-4.60	3.65	< 0.001	3.20-4.16
Positive history of stroke	1.29	< 0.001	1.14-1.47	1.27	< 0.001	1.13-1.43

OR: Odds ratio; CI: Confidence interval; SAH: Subarachnoid hemorrhage; ICH: Intracerebral hemorrhage

The 28 days case fatality rate of Isfahan stroke hospitalized patients was 25.6% and is shown in table 2. Confounding factors’ effects were the same as in hospital case fatality: ICH had the greatest case fatality and case fatality was more in females, elders, and subjects with previous strokes. Furthermore, when all the above variables were put in a logistic regression, stroke subtype was the strongest risk factor, although sex remained significant.

Table 4 shows functional disability of survivors and its associated factors. Of all the patients who were alive after stroke at 28^th^ day, only 26.9% had mRS ˂ 3 (Table 4). The outcome was affected by stroke subtype and the previous history of stroke; about 50.0% of SAH subjects were functionally independent (mRS < 3), while only 15.0% of subjects with the previous stroke were. Male subjects had somehow better functionality than female ones. Moreover, patients with mRS < 3 were in average 7 years younger than patients with mRS ≥ 3. All the above variables were put in a logistic regression to see which one explains more of the variance in the functional independence; stroke history was the most powerful followed by stroke subtype (ICH) (Table 5).

Among subjects who were functionally independent before their strokes, 34.2% still had mRS < 3 on the 28^th^-days after the strokes. SAH patients had the best outcomes; with 60.0% of alive held mRS ˂ 3 on day 28 after their strokes, followed by ischemic (34.5%) and ICH (27.8%) ones (P < 0.001).

Table 6 shows the distribution of sex and age in the study sample in comparison with one study performed in Brazil and another in the USA with the same methodology of patient recruitment as ours.

## Discussion

In this study, 9487 patients with stroke were enrolled from Isfahan’s hospitals, over a period of 5-year. In hospital, case fatality rate in this study was 12.5% for ischemic and 33.9% for ICH stroke subtype which were considerably more than a similar study conducted with 56969 patients enrolled in the USA, yielding an overall case fatality rate of 6.8%, 5.7% case fatality for IS, and 22.7% case fatality for hemorrhagic strokes.^18^ On the contrary, our findings had a resemblance to a recent study which was done with 2407 patients evaluated in Brazil. The overall in hospital case fatality rate was 20.9%, IS case fatality rate was 17.0%, and ICH case fatality rate was 34.1%.^19^ The stroke subtype 28 days case fatality in our study reached 21.2% for IS and 44.8% for ICH. These percentages can be compared to the 28 days case fatality rates in low and middle-income countries classified by the World Bank for IS at 16.7% and ICH at 38.7%.^20^ Although about 17.0% of our patients were lost in their follow-ups, there was a little clinically significant difference between groups with successful and unsuccessful follow-ups: for example, there was only 2.7% difference in missing rate between ICH and ischemic patients. Furthermore, in hospital and 28 days death variables that had different missing proportions (in hospital death had 0.5% missing data while 28 days death had 17.0% missing data) had nearly the same odds ratios with presumed risk factors in logistic regressions. This is another piece of evidence that missing in our study might be non-biased and did not affect our results. 

**Table 4:** Potential confounding factors’ effect on functional outcome at 28^th^ day among Isfahan stroke hospitalized patients, from 2006 to 2011

**Variable**	**mRS ** **˂** ** 3 (total)**	**P**	**mRS ** **˂** ** 3 (if pre-stroke mRS ** **˂** ** 3)**	**P**
All stroke subtypes [n (%)]	1573 (26.9)	< 0.001	1569 (34.2)	< 0.001
Ischemic	1339 (27.4)		1335 (34.5)	
ICH	157 (20.6)		157 (27.8)	
SAH	51 (49.5)		51 (60.0)	
History of previous stroke [n (%)]		< 0.001		
Negative	1309 (30.8)			
Positive	230 (15.6)			
Sex [n (%)]		0.001		
Male	886 (28.7)			
Female	683 (24.9)			
Age (year) (mean ± SD)		< 0.001		
mRS ˂ 3 (n = 1569)	62.62 ± 14.59			
mRS ≥ 3 (n = 4262)	70.09 ± 12.38			

SD: Standard deviation; mRS: Modified Rankin Scale; ICH: Intracerebral hemorrhage; SAH: Subarachnoid hemorrhage

**Table 5:** Multiple logistic regressions of functional independence (MRS < 3) at day 28 among survivors of stroke

**Variable**	**In hospital case fatality**		
	**OR**	**P**	**95% CI of OR**
Age (centered on 70)	0.96	< 0.001	0.96-0.97
Male sex	1.29	< 0.001	1.14-1.46
Type of stroke		< 0.001	
SAH versus ischemic	1.28	0.250	0.84-1.95
ICH versus ischemic	0.52	< 0.001	0.43-0.64
Positive history of stroke	0.45	< 0.001	0.38-0.52

OR: Odds ratio; CI: Confidence interval; SAH: Subarachnoid hemorrhage; ICH: Intracerebral hemorrhage; mRS: Modified Rankin scale

**Table 6:** Comparison of Isfahan hospitalized patients with stroke, from 2006 to 2011, with Brazil's and USA’s

**Variable**	**Brazil**	**USA**	**Iran**
Age (mean ± SD)			
Total	67.70 ± 14.40	69.6 ± 0.1	68.98 ± 13.63
Ischemic	69.14 ± 13.60	-	69.77 ± 13.12
ICH	62.70 ± 14.90	-	66.75 ± 14.52
Sex (female) (%)			
Total	51.8	53.3	48.3
Ischemic	50.1	-	48.2
ICH	47.5	-	48.4
Previous history of stroke (%)			
Total	42.9	-	18.2
Ischemic	46.2	30.7	-
ICH	33.3	20.3	-

ICH: Intracerebral hemorrhage

This greater in hospital and 28^th^ day case fatality rate in our study relative to developed countries could be due to differences in stroke severity in admission. However, it could reflect using low-quality standards for the care of patients with acute stroke. These may include significant delays in hospital admission, diagnosis, and evaluation with neuroimaging, the absence of thrombolytic use for ISs, and lack of stroke units for treatment of patients.

Table 6 shows our study sample sex and age distribution in comparison with Brazil’s and USA’s, which are nearly the same.^19^^,^^20^ This excludes confound effect of age and sex in the differences seen among the countries. Since the factor of the previous history of stroke was significantly less frequent in our study, it could not also explain the greater case fatality observed in Iran. The fact that hospitalized patients were a source of recruitment in the three studies excludes a difference in study methodology (population-based vs. hospital-based) as a potential explanation for studies’ findings. 

Independent factors associated with early functional outcome were investigated using the MRS (range 0-6, 6 denotes death and was excluded for this analysis). This analysis demonstrated five factors: ICH stroke subtype, pre-stroke disability/dependency, positive history of previous stroke, older age and female sex, which were strongly associated with a poor functional outcome (MRS   3) at the 28^th^ day post stroke. Furthermore, another factor explaining this finding might be the differences in medical care in Iran, which are different than in the most developed countries. Indeed, none of our study patients were managed in a stroke unit which might contribute to an increased case fatality and poor functional outcome, and hence necessitate the use of early interventions. Stroke units are a highly evidence-based approach shown to improve outcomes after stroke. A systematic meta-analyses investigating stroke units showed 18.0% reduction in the risk of case fatality and dependency.^20^ One of the major limitations to the implementation of stroke units in Iran is the restricted availability of stroke specialists as well as technical and financial limitations. Other possible obstacles are a deficiency in health resources, considering stroke as not being a prior health problem, and inadequate health personnel training.

Strengths of our study are prospective collection of data from all hospitals in Isfahan, successful follow-up in more than 83.0% of patients and acquisition of functional status in the follow-up. However, our study is a non-population-based one, and it did not cover patients with stroke who were not hospitalized. Other limitations were the use of hospital records to get patients’ history and risk factor profiles, and lack of magnetic resonance imaging for all possible ISs with normal CT scans.

## Conclusion

The global impact of stroke in Iran, similar to other low and middle-income countries, is taking a disproportionate toll on its people. This study of 9487 patients with stroke from Isfahan’s hospitals highlights the great early case fatality and disability due in part to the lack of stroke units. Standards of care were limited by lack of local resources and evidence. Opportunities for simple, inexpensive interventions to improve outcomes or reduce recurrent stroke and case fatality should be identified. In future work, we hope to facilitate implementation of widely accepted, and locally feasible, standards of care through building stroke units that have the potential to reduce the burden of stroke in Iran and other neighboring nations facing this problem.

## Notes:

**How to cite this article:** Oveisgharan Sh, Ghaemmaghami AB, Bahonar A, Sarrafzadegan N. Case fatality and disability of stroke in Isfahan, Iran: Isfahan Stroke Registry. Iran J Neurol 2016; 15(1): 9-15.
